# Oculomics and the NHS: A UK opportunity to translate eye-derived biomarkers into population health

**DOI:** 10.1038/s41433-026-04693-w

**Published:** 2026-07-03

**Authors:** Alexander C. Heatley, Pearse A. Keane, Siegfried K. Wagner

**Affiliations:** 1 Institute of Ophthalmology, University College London, London, UK; 2 NIHR Biomedical Research Centre, Moorfields Eye Hospital NHS Foundation Trust & UCL Institute of Ophthalmology, London, UK

**Keywords:** Prognosis, Biomarkers

The idea that the eye offers a window into systemic health is not new, but the scale, resolution and computability of ophthalmic imaging are unprecedented. Optical coherence tomography (OCT) and fundus photography are embedded in eye care, generating high-dimensional data that capture neural, vascular and structural biology in vivo. Linked to indices of systemic health and longitudinal outcomes, and analysed with modern machine learning, these data underpin oculomics: the use of ophthalmic biomarkers to detect, characterise or predict systemic disease [1, 2]. This commentary suggests that the UK is uniquely placed to translate oculomics from retrospective association to clinically useful prediction, but success will depend less on algorithmic novelty than on governance, prospective validation, implementation and multidisciplinary, cross-sector collaboration to define its place within clinical pathways.

Fundus and OCT-derived structural, vascular and AI phenotypes have been linked to cardiovascular, neurodegenerative, metabolic and renal disease [2]. Poplin and colleagues showed that fundus images encode cardiovascular signal, while framing the findings as hypothesis-generating rather than clinically deployable [3]. More recent work has extended this proof-of-concept into foundation models trained on millions of retinal images and validated for ocular and systemic disease prediction [4]. Reviews describe a growing evidence base, but underscore the need for prospective validation, external generalisability and clinical utility before entering patient care [2, 5].

The UK is well placed to deliver those prerequisites: a tax-funded national health service with longitudinal records, ophthalmic imaging delivered at population scale, maturing governance for secure access to linked health data, decades of experience in delivering screening programmes, and a primary care optometry network through which a substantial proportion of eye care is delivered.

First, the UK captures eye data at scale. It has robust infrastructure for primary care optometry and population-scale diabetic eye screening. Independent community optometry practices deliver large volumes of eye examinations; in England, 5622 GOS contractors provided 12.94 million NHS sight tests in 2022–2023 [6]. The NHS Diabetic Eye Screening Programme invites people with diabetes aged 12 years and over, with screening intervals determined by risk and previous findings [7]. NHS England reported in 2024 that around four million people were registered with the programme, and that 3.3 million undergo screening every one or two years [8]. The announcement described the rollout of OCT in community settings, including larger GP practices, community hospitals and mobile vans, for approximately 60 000 higher-risk patients. Without imaging infrastructure on this scale, no oculomic model will progress beyond retrospective association.

Second, the UK hosts datasets linking ocular imaging with health phenotypes. UK Biobank contributes retinal imaging and OCT from approximately 68,500 participants linked to longitudinal health data [9], while AlzEye links Moorfields ophthalmic imaging with national Hospital Episode Statistics, capturing disease diversity and clinical complexity [10]. SCONe extends this model into Scottish community optometry, linking over 367,000 fundus photographs from more than 36,000 patients to healthcare records [11]. FORESIGHT will complement these, recruiting healthy volunteers through community optometry practices, with the ambition of reaching half a million participants [12].

Third, the UK is developing governance structures designed to make ophthalmic data usable for research while maintaining public trust. INSIGHT, the Health Data Research Hub for Eye Health and Oculomics, is an NHS-led initiative that has grown into the world’s largest ophthalmic bioresource, comprising more than 30 million retinal images linked to clinical records from approximately two million patients [13, 14]. A trusted real-world data resource requires curation, anonymisation, governance, patient and public involvement, and a clear orientation towards patient benefit [14]. UKRI MRC and NIHR funding awarded in November 2025 is expanding INSIGHT from a single-site resource into a national programme, onboarding additional NHS sites, broadening data linkages and integrating genetic data from the NIHR BioResource and UK Biobank, with explicit emphasis on patient involvement, AI bias and equity [15].

The risk is that algorithmic enthusiasm outpaces clinical pathway design. A retinal prediction of cardiovascular, renal or neurodegenerative risk may generate anxiety, unnecessary investigation and medicolegal uncertainty unless the pathway for communicating, confirming and acting on that risk is defined. Comparable ophthalmic cohorts exist internationally, but the UK’s combination of universal coverage and governed access to linked outcomes remains rare.

The questions that matter are not computational but clinical, and four are central. The first is clinical utility: does a positive oculomics prediction benefit patients? This is important in community optometry, where prediction may identify people who would not otherwise have entered risk-stratification pathways. The second is equitable performance: does it perform consistently across ethnic, socioeconomic and disease subgroups, and where it does not, how is that disclosed and mitigated? The third is health economics: do the downstream investigations and workload generated by an oculomic prediction deliver value at acceptable cost within finite NHS budgets? The fourth is pathway development: which clinician owns the result, what is the onward referral route, and how does the prediction integrate with existing services?

Ophthalmologists and optometrists should shape this field, not wait to evaluate tools built elsewhere on datasets that may not reflect NHS populations or pathways. Eye care professionals understand image quality, artefact, media opacity, high myopia and the realities of high-volume NHS eye care, and are well placed to identify where oculomic tools could function as decision support within safe, auditable pathways. They sit at the front line of the patient-facing relationships of trust on which engagement with data-driven research depends.

Four priorities follow. Prospective validation in representative NHS populations. Transparent reporting of model performance, including subgroup analyses and failure modes. Patient and public involvement in data access, consent models and acceptable use cases. And implementation studies that measure accuracy alongside clinical utility, cost-effectiveness, workload and equity.

Oculomics is framed as using the eye as a window onto the body. The more consequential question for the UK is whether we can build the architecture around that window, the governance, datasets, workforce and clinical pathways that turn imaging signals into decisions clinicians can act on and patients can trust. The infrastructure is in place. The next decade is about what we do with it.
